# Strategies to Prevent or Reduce Gender Bias in Peer Review of Research Grants: A Rapid Scoping Review

**DOI:** 10.1371/journal.pone.0169718

**Published:** 2017-01-06

**Authors:** Andrea C. Tricco, Sonia M. Thomas, Jesmin Antony, Patricia Rios, Reid Robson, Reena Pattani, Marco Ghassemi, Shannon Sullivan, Inthuja Selvaratnam, Cara Tannenbaum, Sharon E. Straus

**Affiliations:** 1 Li Ka Shing Knowledge Institute, St. Michael’s Hospital, Toronto, Ontario, Canada; 2 Epidemiology Division, Dalla Lana School of Public Health, University of Toronto, Toronto, Ontario, Canada; 3 Institute of Gender and Health, Canadian Institutes of Health Research, Montréal, Quebec, Canada; 4 Department of Geriatric Medicine, University of Toronto, Medical Sciences Building, Toronto, Ontario, Canada; McGill University, CANADA

## Abstract

**Objective:**

To review the literature on strategies implemented or identified to prevent or reduce gender bias in peer review of research grants.

**Methods:**

Studies of any type of qualitative or quantitative design examining interventions to reduce or prevent gender bias during the peer review of health-related research grants were included. Electronic databases including MEDLINE, EMBASE, Education Resources Information Center (ERIC), PsycINFO, Joanna Briggs, the Cochrane Library, Evidence Based Medicine (EBM) Reviews, and the Campbell Library were searched from 2005 to April 2016. A search for grey (i.e., difficult to locate or unpublished) literature was conducted and experts in the field were consulted to identify additional potentially relevant articles. Two individuals screened titles and abstracts, full-text articles, and abstracted data with discrepancies resolved by a third person consistently.

**Results:**

After screening 5524 citations and 170 full-text articles, one article evaluating gender-blinding of grant applications using an uncontrolled before-after study design was included. In this study, 891 applications for long-term fellowships in 2006 were included and 47% of the applicants were women. These were scored by 13 peer reviewers (38% were women). The intervention included eliminating references to gender from the applications, letters of recommendations, and interview reports that were sent to the committee members for evaluation. The proportion of successful applications led by women did not change with gender-blinding, although the number of successful applications that were led by men increased slightly.

**Conclusions:**

There is limited research on interventions to mitigate gender bias in the peer review of grants. Only one study was identified and no difference in the proportion of women who were successful in receiving grant funding was observed. Our results suggest that interventions to prevent gender bias should be adapted and tested in the context of grant peer review to determine if they will have an impact.

## Introduction

Despite parity between the number of women and men completing undergraduate and graduate training in biomedical and health sciences worldwide [1, 2], women continue to be underrepresented as researchers in these domains and tend to receive less research funding than their male counterparts [3–5]. For example, an analysis of health services and policy research funding in Canada over the past decade found that female researchers under the age of 45 years had significantly lower success rates than age-matched male researchers [6]. Results from the 2015 2^nd^ Pilot competition at the Canadian Institutes of Health Research (CIHR) indicated that mid-career and senior-career female researchers were less likely to be funded in the newly launched Foundation Grant program, which emphasizes track record and provides up to 7 years of funding for the pursuit of potentially high-impact research programs [7]. A similar study of the National Institutes of Health (NIH) in the United States has shown that women received larger individual grant awards but men held more grants than women at any point in their careers [5].

It is unclear whether the discrepancy between the rate of successful male and female grant applicants is a sign of systemic bias. It is important to consider if there is unconscious gender bias in the grant peer review process because career advancement in academic settings is often contingent on the ability to obtain research funds. An unconscious bias is an implicit attitude, stereotype, motivation, or assumption that can occur without one’s knowledge, control, or intention [8]. Forms of unconscious bias include gender bias, racial bias, and ageism, with gender bias representing one of the most frequently investigated biases associated with grant peer review [9–23].

In a study conducted in Sweden, female applicants had to be 2.5 times more productive (in terms of higher volume of publications or publication in journals with a higher impact factor) than male applicants in order to achieve the same “competence” scores on their grant applications [24]. A 2015 study identified gender bias in the peer review process through a linguistic analysis of NIH R01 reviewer comments on applications between 2007 and 2009. They found that successful applications submitted by women received more positive descriptors and praise. Furthermore, experienced female investigators received more references to competence than their male counterparts. Nonetheless, female applicants received similar scores to male applicants, despite the discrepancies in word choice used by the reviewers [25]. In a subsequent study of NIH R01 applications between 2010 and 2014, Kaatz et al. 2016 reported that despite more standout adjectives (e.g., outstanding) and references to ability being used in female applications than male applications, peer reviewers were more likely to assign statistically significantly worse priority, approach, and significance scores to female than male investigators [26].

In response to these concerns of inequity in the grant review process, several funders have implemented strategies to narrow the gender gap. In the United States, the NIH has implemented programmatic strategies to promote and support careers of women in biomedical science such as changing the wording in their grant announcements to ensure gender neutrality and establishing strategies to monitor equity across grant programs [27, 28]. However, their impact is unclear because these programs were delivered at the same time that individual universities were making attempts to address gender bias [29]. In the United Kingdom, the National Institute for Health Research made funding contingent on the candidate organizations receiving at least a Silver Award from the Athena Scientific Women’s Academic Network (SWAN) Charter; an award that signifies institutional attempts to advance gender equality [30]. To inform efforts at the CIHR, we completed a rapid scoping review of strategies to prevent or reduce gender bias in the peer review of research grants.

## Methods

We developed a protocol using the scoping review methods proposed by Arksey and O’Malley [31] and further refined by the Joanna Briggs Institute [32]. To provide the CIHR with a timely answer in less than 2 months, we completed a rapid review [33]. This review was registered through the Open Science Framework (https://osf.io/qdzpt/). Although the PRISMA statement [34] has not been modified for scoping reviews, we used it to guide our reporting where possible (S1 Appendix).

### Eligibility criteria

Our eligibility criteria were defined using ‘Population, Intervention, Comparison, Outcomes, Study designs, Timeframe’ (PICOST) components, with input from the knowledge user (CT).

### Population

Peer reviewers (including researchers or knowledge users) or grant applicants for any type of research grants (e.g. clinical, health services, education) and any sex/gender. For descriptive studies, the participants were those who submitted a grant proposal to peer review.

### Intervention

Any strategy to prevent or decrease gender bias in the peer review of research grants.

### Comparators

Any other intervention or no intervention; studies without a comparator were also eligible for inclusion.

### Outcomes

Any outcome that assessed or measured gender bias (including awareness, knowledge, attitudes) or its potential impact (such as proportion of funded research projects by women).

### Study designs

All qualitative or quantitative study designs.

### Timeframe

Due to the rapid nature of this review, we restricted the timeframe to papers published from 2005 to April 2016.

### Other

We limited documents to those published in English due to the rapid nature of this review.

### Information sources and search strategy

The protocol for comprehensive literature searches were developed by an experienced information specialist (JM) in consultation with the research team and completed by a library technician (AE). First, we searched MEDLINE, EMBASE, Education Resources Information Center (ERIC), PsycINFO, Joanna Briggs, the Cochrane Library, Evidence Based Medicine (EBM) Reviews, and the Campbell Library from 2005 to April 2016. Second, we searched for grey literature (i.e. difficult to locate or unpublished material) using the Canadian Agency for Drugs and Technologies in Health Grey Matters checklist [35]. Specifically, we searched Google and websites of funding agencies such as the CIHR, UK MRC and NIH. Third, we asked experts in the field to identify any additional potentially relevant articles. We limited the search to articles published in English. The final search strategy for MEDLINE is presented in S2 Appendix and additional search strategies are available from the corresponding author upon request.

### Study selection

Search results were imported into our proprietary software for screening citations (i.e. titles and abstracts) and full-text articles [36]. The inclusion criteria were also imported into this online software and used for screening citations during the screening of titles and abstracts (i.e., level 1 screening) and full-text articles (i.e., level 2 screening).

We completed a series of calibration exercises prior to each stage of screening to ensure reliability across reviewers. Inter-rater agreement for study inclusion was calculated using percent agreement and when it reached >75% across the research team, we proceeded to the next stage. If the percent agreement was ≤75%, the inclusion criteria were clarified and another pilot test occurred. For level 1 screening (title and abstracts), one pilot test of 50 citations was conducted with all team members and we achieved 92% agreement. Subsequently, two reviewers independently reviewed all titles and abstracts for inclusion. For full-text screening, one pilot test of 13 full-text articles was conducted with all team members, and we achieved 85% agreement. Following this calibration exercise, two reviewers screened the full-text of potentially relevant articles to determine inclusion. The results of the grey literature search were initially screened by a single reviewer, and the full-text of any potentially relevant grey literature identified was then assessed by two reviewers. All discrepancies between reviewers were resolved by a third reviewer consistently (SMT or JA).

### Data collection

We abstracted data on study characteristics (e.g. year of study conduct, country, setting, type of publication, focus of the study), population characteristics (e.g. % female, % new investigators), and quantitative (e.g. % successful applicants) outcomes. Due to the small number of included studies identified, a pilot-test was not conducted for data abstraction. The data abstraction form was developed and modified as required based on feedback from the team. Each study was abstracted by two team members and any discrepancies were resolved by a third person (SMT).

### Methodological quality appraisal

We did not appraise quality or risk of bias of the included articles, consistent with accepted scoping review methods [31, 32] and scoping reviews on health-related topics [37].

### Synthesis and data charting

We charted the data quantitatively to identify the number of relevant publications according to types of participants, interventions, comparators, and outcomes, and summarized these findings using descriptive frequencies. We obtained additional data from the authors of one study and included this information in the analysis [38].

### Consultation

We provided the results to the CIHR knowledge user (CT) as well as 3 experts in the field of gender bias for their review. Their comments were used to inform the presentation of our results. Patients were not engaged with the conception or conduct of this review.

## Results

### Literature search

**Fig 1:**
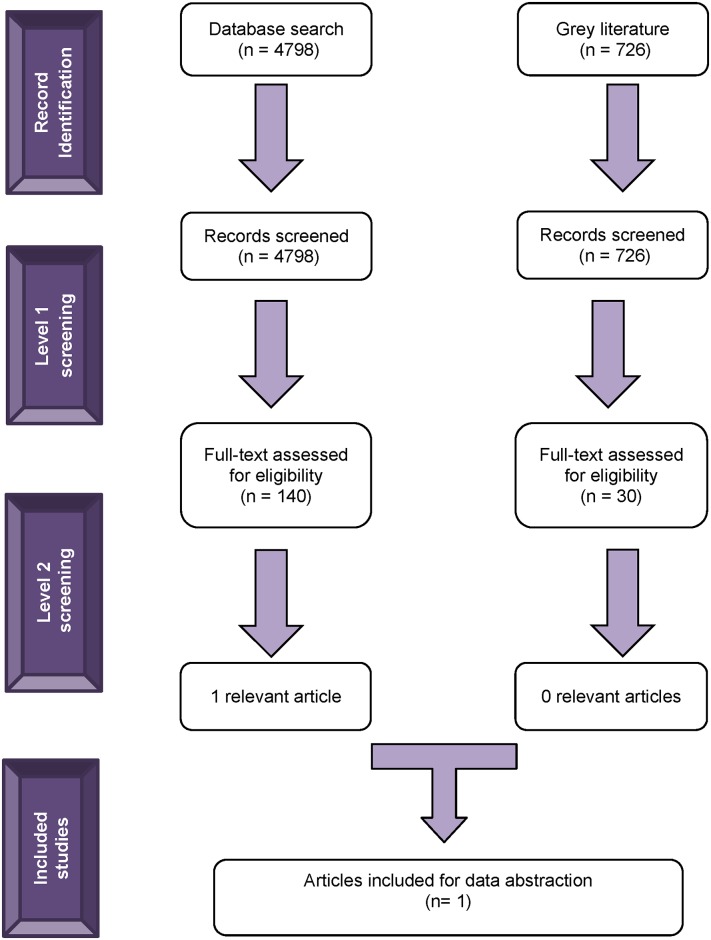
Study Flow Diagram. Breakdown of the number of studies identified in the literature, assessed for eligibility, and finally included in the rapid scoping review.

We retrieved 4,798 citations from the electronic database search (Fig 1). Of these, 140 citations were potentially relevant and their full-texts were reviewed. Subsequently, 1 article met our eligibility criteria from the database search and was included [38]. We identified 726 records from the grey literature search. Of these, 30 records were potentially relevant and their full-texts were reviewed, however none of these were deemed to be relevant for inclusion from the grey literature search [39–41].

### Publication and participant characteristics

The included study was an uncontrolled before-after study conducted in Europe examining gender bias using an intervention [38]. The gender-blinding experiment was briefly described in Ledin 2007 [38], and additional details were obtained by contacting the corresponding author (S3 Appendix). For this study, 891 applications to the European Molecular Biology Organization (EMBO) Long-Term Fellowship programme were sent to 13 peer reviewers to be scored, after all references to the applicant’s gender had been removed. Forty-seven percent of the grant applicants, and 38% of the peer reviewers were female.

### Interventions to potentially mitigate gender bias in peer review

Ledin and colleagues (2007) assessed the success of female applicants before (i.e. 2002 to 2005) and after (i.e. 2006) gender-blinding of the applications [38]. A detailed description of their intervention and data is provided in the S3 Appendix. From 2002 to 2005, 14.8% of applications led by women were successful compared with 18.1% of applications led by men (S3 Appendix). In 2006, they eliminated references to gender from the applications, letters of recommendations, and interview reports that were sent to the committees for scoring. The proportion of applications that were successful and led by women did not change after gender blinding (14.7%) although there was a slight increase in the successful applications that were led by men (20.7%). Given that lists of publications were provided by the applicants, there was nothing prohibiting the peer reviewers from conducting an internet search to identify the applicant’s identity, however the study authors felt that this was unlikely to occur given the number of applications each reviewer was assigned. Nonetheless, because it was perceived that gender blinding of the applications was not effective, this strategy was discontinued in 2007. From 2007 to 2014, there was a slight decrease in the proportion of funded applications that were submitted by women (13.8%).

## Discussion

Several studies, conducted internationally, have identified that women are less likely than men to be funded in research grant competitions and this has not changed substantively over time [5–7, 38, 42, 43]. We identified only one study that assessed a strategy (gender-blinding of applications) to mitigate the risk of gender bias in grant peer review. The results of this study were negative [38]. However, it was uncontrolled and reviewers may have drawn conclusions about the applicants based on the de-identified information provided, including lists of prior publications. As well, some studies have found that letters of recommendation for men had a greater proportion of standout adjectives (e.g., excellent, superb, outstanding, unique) than women [44], which may also have influenced the results of the Ledin et al. study. Other recommendations to mitigate gender bias in peer review may entail ensuring appropriate representation of men and women on peer review panels, training peer reviewers in unconscious bias, and ensuring evaluation criteria are consistently applied [40, 43]. Self-awareness and scrutiny of the language and descriptors used to qualify under-represented populations may be effective for gender stereotypes. These recommendations are limited by a lack of studies that have evaluated their true impact.

Other fields in academia outside of grant peer review have likewise explored gender bias and potential strategies to minimize it, and this work can inform future steps in improving grant peer review within science. For example, a 2009 systematic review of interventions that impact gender bias in hiring examined employment for all types of positions and found a negative bias towards women being evaluated for positions that are traditionally or predominantly held by men [45–47]. This earlier review identified strategies to mitigate this bias that could be considered potentially relevant to grant peer review, including using structured evaluations versus unstructured evaluations and implementing training workshops to make peer reviewers aware of common biases and how to overcome such biases [45]. A more recent study found that a 20-minute workshop providing education on implicit biases and strategies for overcoming them changed participants’ perceptions of bias; this type of workshop could be considered for grant peer reviewers [46]. In another relevant study, a cluster-randomised trial of faculty from 92 departments (including medicine) at one university showed an increase in self-efficacy to engage in gender-equity promoting behaviours following a 2.5 hour workshop [47]. When more than 25% of department members attended this workshop, there was an increase in self-reported activity to promote gender equity at 3 months [47]. Furthermore, in contrast to the uncontrolled before-after study included in our review, anonymous review of applicants without disclosing the investigator’s name or gender was found to be effective in other settings [48], suggesting that it might be useful to test this strategy using a more rigorous design, such as in symphony orchestras and the technology industry [49–51]. Finally, when preparing peer review reports, peer reviewers may wish to ensure that they spend equal time on positive and negative aspects of the grant [52] and that they are aware of potential unconscious bias on gender differences [44].

There are several limitations to our rapid scoping review that should be considered. First, our search was limited to the period from 2005 to 2016 because it was a rapid review. As a result, we may have missed publications that might have contributed meaningfully to our findings. However, our discussions with experts in the field suggested that we did not miss any large, landmark studies published before 2005 that may have influenced our results. Second, we limited the articles to those published in English for feasibility reasons. Most of the studies were conducted in high-income countries similar to Canada, highlighting the fact that the results are applicable to our national funding agency, CIHR. Third, because this was a scoping review, we did not conduct a risk of bias assessment of the included study.

We believe a high quality systematic review of the literature focusing on exploring the potential for gender differences in research grant applications also be considered, given this is currently lacking. And if this systematic review indicates that a prospective study is necessary, we would encourage funding agencies to fund an experimental study. In particular, studies of NIH data show mixed results [10–13], while data from the Wellcome Trust and UK MRC show no difference [14] in success rates between male and female applicants. Similarly, data from the Australian Research Council [15] did not show a difference in success rates. Data from the European Molecular Biology Organisation showed female applicants had lower success rates than men over the period 1996–2001 [16]. The European Council Research grants found women had a lower success rate than male applicants from 2008–2013 [17]. Data from the Netherlands Organisation for Scientific Research showed evidence of gender differences in success rates [18]. Data from the landmark Swedish Medical Research Council showed a gender gap [19]; Hallsten [20] conducted a review of data from the sub-council for Medicine within the Swedish Research Council and found that applications from men without an affiliation with a reviewer received lower scores than female applicants. A recent unpublished study shows that women were less successful than men in receiving funding from European Research Council grants, even after for controlling for factors such as the number of publications and grants received by the applicant [43]. And, several studies from various countries have shown that on average, women receive fewer and smaller grants than men; the reasons for this are not clear [4, 21, 22].

## Conclusion

This review represents the first rapid scoping review of gender bias in grant peer review. It has highlighted substantial gaps in this area and the need for funding agencies to evaluate the impact of initiatives they implement to mitigate gender bias in peer review. Randomised trials of different training strategies for grant peer reviewers including, completion of the Harvard Implicit Association Test or participation in online education about how to break gender stereotyping and promote gender equity, may help strengthen the evidence for granting agencies to change current practice. Furthermore, the implementation of the Athena SWAN initiative in the UK provides an opportunity to explore its impact on grant success of female researchers. Recent data from a realist evaluation show that the Athena SWAN program is perceived to have an important impact across participating universities but is associated with a heavy workload to fulfil its requirements. Further complicating this issue is that the workload is largely being shouldered by women, unintentionally reproducing gender inequity through its very enactment [53]. Establishing best practices for grant peer review to reduce gender bias will benefit from more supporting evidence. Active strategies are needed to address potential gender bias in grant peer review to ensure that the creativity and innovation offered by our diverse population is not lost.

## Supporting Information

S1 Appendix PRISMA Checklist. (PDF)

S2 Appendix Search Strategy. (PDF)

S3 Appendix Detailed description of the gender-blinding intervention and additional data. (PDF)
