# Supplementary material for: Strategies to Prevent or Reduce Gender Bias in Peer Review of Research Grants: A Rapid Scoping Review
Source: PLoS One. 2017 Jan 6;12(1):e0169718. doi: 10.1371/journal.pone.0169718 (PMC5218731; doi:10.1371/journal.pone.0169718)
Supplement: S2 Appendix — (PDF) [file pone.0169718.s002.pdf]

Appendix 2. MEDLINE search strategy

Database: Ovid MEDLINE(R) In-Process & Other Non-Indexed Citations and Ovid MEDLINE(R) <1946 to Present>

Search Strategy:

----

1 exp "Peer Review"/

2 (peer review* or peer-review* or ethical review* or referred or refereeing or grant review*).tw.

3 1 or 2

4 Financing, Organized/

5 exp Training Support/

6 "Awards and Prizes"/

7 Research Support as Topic/

8 (grant or grants or award or awards or financial support or fund* support or prize or prizes or research support or research fund*).tw.

9 or/4-8

10 3 and 9

11 limit 10 to english

12 limit 11 to yr="2005 -Current"
