# Supplementary material for: Strategies to Prevent or Reduce Gender Bias in Peer Review of Research Grants: A Rapid Scoping Review
Source: PLoS One. 2017 Jan 6;12(1):e0169718. doi: 10.1371/journal.pone.0169718 (PMC5218731; doi:10.1371/journal.pone.0169718)
Supplement: S3 Appendix — (PDF) [file pone.0169718.s003.pdf]

Appendix 3. Description of the gender-blinding intervention and additional data

Ledin et al. (2007) described using gender-blinding during one submission cycle of the European Molecular Biology Organization's (EMBO) Long-Term Fellowship in 2006 [1]. They reported that this had no effect in reducing the gender gap, so the gender-blinding was not used in subsequent years. We contacted the corresponding author to obtain additional information on how gender-blinding was implemented.

Author's description of how the intervention was implemented:

"We eliminated all references to gender from the applications, letters of recommendation and interview reports that were sent to the committee for scoring. Specifically:

1. We replaced the first name with the initials in the application and the letters of reference.

2. We removed any indication of gender in the letters of reference by replacing he or she with he/she and her or his with her/his. Same for the interview report.

3. Our selection procedure involves a personal interview with an EMBO Member expert in the area of research of the applicant. Obviously we could not gender blind the interviewer, but removed any reference to gender in the report as describe above. To test whether the reports were written in such a way that gender was obvious (as described for example in the paper by Trix and Penska: the colour of glass) several of us read all (gender blinded) reports and tried to guess that gender of the applicant from the way the report was written. Of the 280 reports that we read, we ventured guesses in 50 cases, of which 50% of our guesses were correct. From this we concluded that gender could not be inferred from the reports themselves.

4. Given the publications (first names were indicated by an initial) the committee members could have looked the person up and find out their gender. Since each committee member looks at over 180 applications they usually do not take the time to do that. But of course, that is a caveat."

Additional data provided by author:

EMBO Long-Term Fellowships

	Applications	%	Awards	%	Success rate %
**2002**					
Female applicants	347	43	68	41	20
Male applicants	453	57	100	59	22
Overall	800		168		21
**2003**					
Female applicants	469	43	64	40	14
Male applicants	611	57	112	60	18
Overall	1080		138		16
**2004**					

Female applicants	533	47	65	40	12
Male applicants	604	53	98	4860	16
Overall	1137		163		14
**2005**					
Female applicants	589	48	89	45	15
Male applicants	647	52	109	55	17
Overall	1236		198		16
**2006**					
Female applicants	593	48	87	40	15
Male applicants	644	52	133	60	21
Overall	1237		220		18
**2007**					
Female applicants	582	45	82	39	14
Male applicants	706	55	130	61	18
Overall	1288		212		17
**2008**					
Female applicants	590	48	81	38	14
Male applicants	628	52	134	62	21
Overall	1218		215		18
**2009**					
Female applicants	657	49	115	45	18
Male applicants	675	51	143	55	21
Overall	1332		258		19
**2010**					
Female applicants	802	48	123	45	15
Male applicants	866	52	152	55	18
Overall	1668		275		17
**2011**					
Female applicants	748	46	103	38	14
Male applicants	885	54	165	62	19
Overall	1633		268		16
**2012**					
Female applicants	739	48	115	47	16
Male applicants	816	52	132	53	16
Overall	1555		247		16
**2013**					
Female applicants	793	48	77	42	10
Male applicants	852	52	108	58	13
Overall	1645		185		11
**2014**					
Female applicants	742	45	86	35	12
Male applicants	906	55	161	65	18
Overall	1648		247		15
**2010-2014**					
Female applicants	3824	47	504	41	13
Male applicants	4325	53	718	59	17
Overall	8149		1222		15

Reference

1. Ledin A, Bornmann L, Gannon F, Wallon G. A persistent problem. Traditional gender roles hold back female scientists. EMBO reports. 2007;8(11):982-7. Epub 2007/11/02. doi: 10.1038/sj.embor.7401109. PubMed PMID: 17972895; PubMed Central PMCID: PMCPMC2247380.
